# Supplementary material for: A UMLS-based spell checker for natural language processing in vaccine safety
Source: BMC Med Inform Decis Mak. 2007 Feb 12;7:3. doi: 10.1186/1472-6947-7-3 (PMC1805499; doi:10.1186/1472-6947-7-3)
Supplement: Additional file 13 — RAP application directory. Sets up directory for RAP (RDF application for PHP) source code [file 1472-6947-7-3-S13.gz › rap/doc/phpdoc/model/DbModel.html]

xml version="1.0" encoding="iso-8859-1"?


Docs For Class DbModel


## Class DbModel

Description

Description |
Descendents
| Methods (details)

This class provides methods for manipulating DbModels from DbStore.

A DbModel is an RDF Model, which is persistently stored in a relational database. This Class uses ADOdb Library for PHP V3.60 (http://php.weblogs.com/ADODB).

History:

- access: public
- author: Radoslaw Oldakowski <radol@gmx.de>
- version: V0.9.1

Located in /model/DbModel.php (line 32)

```
Object
   |
   --Model
      |
      --DbModel
```

Direct descendents

Description |
Descendents
| Methods (details)

| Class | Description |
| --- | --- |
| NamedGraphDb | Persistent NamedGraph implementation that extends a DbModel. |

Method Summary

Description |
Descendents |
Methods (details)

DbModel
DbModel
(object ADOConnection &$dbConnection, string $modelURI, string $modelID, [string $baseURI = NULL])

void
add
(object Statement &$statement)

void
addModel
(object Model &$model)

void
addNamespace
(mixed $prefix, mixed $nmsp, String $prefix,)

void
addParsedNamespaces
(Array $newNs)

void
addWithoutDuplicates
(object Statement &$statement)

void
close
()

boolean
contains
(object Statement &$statement)

boolean
containsAll
(object Model &$model)

boolean
containsAny
(object Model &$model)

void
delete
()

boolean
equals
(object model &$that)

object MemModel
find
(object Resource $subject, object Resource $predicate, object Node $object)

integer
findCount
(object Resource $subject, object Resource $predicate, object Node $object)

object Statement
findFirstMatchingStatement
(object Resource $subject, object Resource $predicate, object Node $object, [integer $offset = -1])

object MemModel
findRegex
(string $subject\_regex, string $predicate\_regex, string $object\_regex)

object MemModel
findVocabulary
(string $vocabulary)

object MemModel
&getMemModel
()

Array
getParsedNamespaces
()

object MemModel
&intersect
(object Model &$model)

boolean
isEmpty
()

array
rdqlQuery
(string $queryString, [boolean $returnNodes = TRUE])

object RdqlResultIterator
rdqlQueryAsIterator
(string $queryString, [boolean $returnNodes = TRUE])

object MemModel
&reify
()

void
remove
(object Statement &$statement)

void
removeNamespace
(String $nmsp)

void
replace
(object Resource $subject, object Resource $predicate, object Node $object, object Node $replacement)

boolean
saveAs
(string $filename, [string $type = 'rdf'])

void
setBaseURI
(string $uri)

integer
size
()

object MemModel
&subtract
(object Model &$model)

string
toString
()

string
toStringIncludingTriples
()

object MemModel
&unite
(object Model &$model)

void
writeAsHtml
()

void
writeAsHtmlTable
()

string
writeRdfToString
()

Methods

Description |
Descendents |
Methods (details)

Constructor DbModel (line 75)

Constructor Do not call this directly.

Use the method getModel,getNewModel or putModel of the Class DbStore instead.

- access: public

DbModel

DbModel
(object ADOConnection &$dbConnection, string $modelURI, string $modelID, [string $baseURI = NULL])

- object ADOConnection
  &$dbConnection
- string
  $modelURI
- string
  $modelID
- string
  $baseURI

add (line 137)

Add a new triple to this DbModel.

- access: public
- throws: PhpError
- throws: SqlError

void

add
(object Statement &$statement)

- object Statement
  &$statement

addModel (line 841)

Add the given model to this DbModel.

This function monitors for SQL errors, and will commit if no errors have occured, otherwise it will rollback. If any statement of the model to be added to this model contains a blankNode with an identifier already existing in this model, a new blankNode is generated.

- access: public

void

addModel
(object Model &$model)

- object Model
  $model

addNamespace (line 1136)

Adds a namespace and prefix to the model.

- access: public
- author: Tobias Gau� <tobias.gauss@web.de>

void

addNamespace
(mixed $prefix, mixed $nmsp, String $prefix,)

- String
  $prefix,: String $nmsp

addParsedNamespaces (line 1121)

Adds the namespaces to the model. This method is called by the parser. !!!! addParsedNamespaces() not overwrites manual added namespaces in the model !!!!

- access: public
- author: Tobias Gau� <tobias.gauss@web.de>

void

addParsedNamespaces
(Array $newNs)

- Array
  $newNs

addWithoutDuplicates (line 181)

Alias for the method add()

- access: public
- throws: PhpError
- throws: SqlError

void

addWithoutDuplicates
(object Statement &$statement)

- object Statement
  &$statement

close (line 911)

Close this DbModel

- access: public

void

close
()

contains (line 315)

Check if the DbModel contains the given statement

- access: public

boolean

contains
(object Statement &$statement)

- object Statement
  &$statement

containsAll (line 335)

Determine if all of the statements in the given model are also contained in this DbModel.

- access: public

boolean

containsAll
(object Model &$model)

- object Model
  &$model

containsAny (line 368)

Determine if any of the statements in the given model are also contained in this DbModel.

- access: public

boolean

containsAny
(object Model &$model)

- object Model
  &$model

delete (line 893)

Remove this DbModel from database and clean up.

This function monitors for SQL errors, and will commit if no errors have occured, otherwise it will rollback.

- access: public
- throws: SqlError

void

delete
()

equals (line 706)

Check if two models are equal.

Two models are equal if and only if the two RDF graphs they represent are isomorphic.

Warning: This method doesn't work correct with models where the same blank node has different identifiers in the two models. We will correct this in a future version.

- access: public
- throws: PhpError

boolean

equals
(object model &$that)

- object model
  &$that

find (line 408)

General method to search for triples in the DbModel.

NULL input for any parameter will match anything. Example: $result = $m->find( NULL, NULL, $node ); Finds all triples with $node as object.

- access: public
- throws: PhpError
- throws: SqlError

object MemModel

find
(object Resource $subject, object Resource $predicate, object Node $object)

- object Resource
  $subject
- object Resource
  $predicate
- object Node
  $object

findCount (line 550)

Search for triples and return the number of matches.

NULL input for any parameter will match anything.

- access: public
- throws: PhpError
- throws: SqlError

integer

findCount
(object Resource $subject, object Resource $predicate, object Node $object)

- object Resource
  $subject
- object Resource
  $predicate
- object Node
  $object

findFirstMatchingStatement (line 506)

Search for triples and return the first matching statement.

NULL input for any parameter will match anything. Return an NULL if nothing is found. You can set an search offset with $offset.

- access: public
- throws: PhpError
- throws: SqlError

object Statement

findFirstMatchingStatement
(object Resource $subject, object Resource $predicate, object Node $object, [integer $offset = -1])

- object Resource
  $subject
- object Resource
  $predicate
- object Node
  $object
- integer
  $offset

findRegex (line 456)

Method to search for triples using Perl-style regular expressions.

NULL input for any parameter will match anything. Example: $result = $m->find\_regex( NULL, NULL, $regex ); Finds all triples where the label of the object node matches Return an empty MemModel if nothing is found. !!!!!!!!!!!!!!!!!!!!!!!!!!!!!!!!!!!!!!!!!!!!!!!!!!!!!!!!!!!!!!!!!!!!!! WARNING: Mhis method loads a DbModel into memory and performs the search on a MemModel, which can be slow with large models. !!!!!!!!!!!!!!!!!!!!!!!!!!!!!!!!!!!!!!!!!!!!!!!!!!!!!!!!!!!!!!!!!!!!!!

- access: public
- throws: PhpError
- throws: SqlError

object MemModel

findRegex
(string $subject\_regex, string $predicate\_regex, string $object\_regex)

- string
  $subject\_regex
- string
  $predicate\_regex
- string
  $object\_regex

findVocabulary (line 476)

Return all tripels of a certain vocabulary.

$vocabulary is the namespace of the vocabulary inluding a # : / char at the end. e.g. http://www.w3.org/2000/01/rdf-schema# Return an empty model if nothing is found.

- access: public
- throws: PhpError
- throws: SqlError

object MemModel

findVocabulary
(string $vocabulary)

- string
  $vocabulary

getMemModel (line 243)

Create a MemModel containing all the triples of the current DbModel

- access: public

object MemModel

&getMemModel
()

getParsedNamespaces (line 1097)

Returns the models namespaces.

- access: public
- author: Tobias Gau� <tobias.gauss@web.de>

Array

getParsedNamespaces
()

intersect (line 810)

Return a new MemModel containing all the statements which are in both this model and the given model.

- access: public
- throws: PhpError

object MemModel

&intersect
(object Model &$model)

- object Model
  $model

isEmpty (line 121)

Check if this DbModel is empty

- access: public

boolean

isEmpty
()

rdqlQuery (line 589)

Perform an RDQL query on this DbModel.

This method returns an associative array of variable bindings. The values of the query variables can either be RAP's objects (instances of Node) if $returnNodes set to TRUE, or their string serialization.

- return: [][?VARNAME] = object Node (if $returnNodes = TRUE) OR array [][?VARNAME] = string
- access: public

array

rdqlQuery
(string $queryString, [boolean $returnNodes = TRUE])

- string
  $queryString
- boolean
  $returnNodes

rdqlQueryAsIterator (line 623)

Perform an RDQL query on this DBModel.

This method returns an RdqlResultIterator of variable bindings. The values of the query variables can either be RAP's objects (instances of Node) if $returnNodes set to TRUE, or their string serialization.

- return: = with values as object Node (if $returnNodes = TRUE) OR object RdqlResultIterator = with values as strings if (if $returnNodes = FALSE)
- access: public

object RdqlResultIterator

rdqlQueryAsIterator
(string $queryString, [boolean $returnNodes = TRUE])

- string
  $queryString
- boolean
  $returnNodes

reify (line 879)

Reify the DbModel.

Return a new MemModel that contains the reifications of all statements of this DbModel.

- access: public

object MemModel

&reify
()

remove (line 195)

Remove the given triple from this DbModel.

- access: public
- throws: PhpError
- throws: SqlError

void

remove
(object Statement &$statement)

- object Statement
  &$statement

removeNamespace (line 1183)

removes a single namespace from the model

- access: public
- author: Tobias Gau� <tobias.gauss@web.de>

void

removeNamespace
(String $nmsp)

- String
  $nmsp

replace (line 644)

General method to replace nodes of a DbModel.

NULL input for any parameter will match nothing. Example: $m->replace($resource, NULL, $node, $replacement); Replaces all $node objects beeing subject or object in any triple of the model with the $replacement node. Throw an error in case of a paramter mismatch.

- access: public
- throws: PhpError
- throws: SqlError

void

replace
(object Resource $subject, object Resource $predicate, object Node $object, object Node $replacement)

- object Resource
  $subject
- object Resource
  $predicate
- object Node
  $object
- object Node
  $replacement

saveAs (line 300)

Saves the RDF,N3 or N-Triple serialization of the DbModel to a file.

You can decide to which format the model should be serialized by using a corresponding suffix-string as $type parameter. If no $type parameter is placed this method will serialize the model to XML/RDF format. Returns FALSE if the DbModel couldn't be saved to the file.

- access: public

boolean

saveAs
(string $filename, [string $type = 'rdf'])

- string
  $filename
- string
  $type

setBaseURI (line 92)

Set a base URI for the DbModel.

Affects creating of new resources and serialization syntax.

- access: public
- throws: SqlError

void

setBaseURI
(string $uri)

- string
  $uri

size (line 108)

Return the number of statements in this DbModel

- access: public

integer

size
()

subtract (line 779)

Return a new MemModel that is the subtraction of another model from this DbModel.

- access: public
- throws: PhpError

object MemModel

&subtract
(object Model &$model)

- object Model
  $model

toString (line 217)

Short dump of the DbModel.

- access: public

string

toString
()

---

Redefinition of:

Object::toString()
:   Serializes a object into a string

toStringIncludingTriples (line 229)

Dump of the DbModel including all triples.

- access: public

string

toStringIncludingTriples
()

unite (line 748)

Return a new MemModel that is the set-union the model with another model.

The result of taking the set-union of two or more RDF graphs (i.e. sets of triples) is another graph, which we will call the merge of the graphs. Each of the original graphs is a subgraph of the merged graph. Notice that when forming a merged graph, two occurrences of a given uriref or literal as nodes in two different graphs become a single node in the union graph (since by definition they are the same uriref or literal) but blank nodes are not 'merged' in this way; and arcs are of course never merged. In particular, this means that every blank node in a merged graph can be identified as coming from one particular graph in the original set of graphs.

Notice that one does not, in general, obtain the merge of a set of graphs by concatenating their corresponding N-triples documents and constructing the graph described by the merged document, since if some of the documents use the same node identifiers, the merged document will describe a graph in which some of the blank nodes have been 'accidentally' merged. To merge Ntriples documents it is necessary to check if the same nodeID is used in two or more documents, and to replace it with a distinct nodeID in each of them, before merging the documents. (Not implemented yet !!!!!!!!!!!)

- access: public
- throws: PhpError

object MemModel

&unite
(object Model &$model)

- object Model
  $model

writeAsHtml (line 255)

Write the RDF serialization of the \_DbModel as HTML.

- access: public

void

writeAsHtml
()

writeAsHtmlTable (line 267)

Write the RDF serialization of the DbModel as HTML table.

- access: public

void

writeAsHtmlTable
()

writeRdfToString (line 280)

Write the RDF serialization of the DbModel to string

- access: public

string

writeRdfToString
()

#### Inherited Methods


Inherited From Model

> Model::Model()  
> Model::findForward()  
> Model::getBaseURI()  
> Model::getMemModelByRDQL()  
> Model::getOntModel()  
> Model::getResmodel()  
> Model::load()  
> Model::visualize()

Inherited From Object

> Object::toString()

Documentation generated on Thu, 7 Jul 2005 13:41:21 +0200 by phpDocumentor 1.3.0RC3
